# Efficient Mining and Characterization of Two Novel Keratinases from Metagenomic Database

**DOI:** 10.3390/biom15111527

**Published:** 2025-10-30

**Authors:** Jue Zhang, Guangxin Xu, Zhiwei Yi, Xixiang Tang

**Affiliations:** Key Laboratory of Marine Genetic Resources, Third Institute of Oceanography, Ministry of Natural Resources, Xiamen 361006, China; momozjue@gmail.com (J.Z.); xuguangxin@tio.org.cn (G.X.)

**Keywords:** keratinase, metagenomics, structural prediction, enzymatic characterization

## Abstract

Keratin is a fibrous structural protein found in various natural materials such as hair, feathers, and nails. Its high stability and cross-linked structure make it resistant to degradation by common proteases, leading to the accumulation of keratinous waste in various industries. In this study, we developed and validated an effective bioinformatics-driven strategy for mining novel keratinase genes from the Esmatlas (ESM Metagenomic Atlas) macrogenomic database. Two candidate genes, *ker820* and *ker907*, were identified through sequence alignment, structural modeling, and phylogenetic analysis, and were subsequently heterologously expressed in *Escherichia coli* Rosetta (DE3) with the assistance of a solubility-enhancing chaperone system. Both enzymes belong to the Peptidase S8 family. Enzymatic characterization revealed that GST-tagged ker820 and ker907 exhibited strong keratinolytic activity, with optimal conditions at pH 9.0 and temperatures of 60 °C and 50 °C, respectively. Both enzymes showed significant degradation of feather and cat-hair keratin. Kinetic analysis showed favorable catalytic parameters, including $K_{m}$ values of 9.81 mg/mL (ker820) and 5.25 mg/mL (ker907), and $V_{\max}$ values of 120.99 U/mg (ker820) and 89.52 U/mg (ker907). Stability tests indicated that GST-ker820 retained 70% activity at 60 °C for 120 min, while both enzymes remained stable at 4 °C for up to 10 days. These results demonstrate the high catalytic capacity, thermal stability, and substrate specificity of the enzymes, supporting their classification as active keratinases. This study introduces a promising strategy for efficiently discovering novel functional enzymes using an integrated computational and experimental approach. Beyond keratinases, this methodology could be extended to screen for enzymes with potential applications in environmental remediation.

## 1. Introduction

Keratin is a fibrous structural protein found in a wide range of materials such as hair, feathers, nails, and horns [1]. It is characterized by its highly stable and cross-linked structure, which includes extensive disulfide bonds formed between the thiol groups (–SH) of cysteine amino acid residues, as well as hydrogen bonds and hydrophobic interactions, making it remarkably resistant to chemical and enzymatic degradation [2]. This resilience contributes to keratin’s role as a protective material in various organisms, but it also presents a significant challenge in waste management, as keratin-rich byproducts accumulate in landfills and take years to degrade naturally [3,4].

The persistence of keratin in the environment poses serious waste disposal challenges, particularly in industries such as agriculture, poultry, and textiles, which generate substantial amounts of keratin waste [5]. Traditional methods of disposal, such as incineration or landfilling, not only fail to address the sustainability of keratin waste, but also contribute to pollution and greenhouse gas emissions [6]. Therefore, there is a pressing need to explore biotechnological solutions for keratin degradation, which can offer more sustainable and environmentally friendly alternatives.

Keratinases, a group of proteolytic enzymes capable of degrading keratin, have drawn significant attention due to their potential in various industrial and environmental applications [7]. These enzymes facilitate the breakdown of keratin-rich waste, offering an eco-friendly alternative to traditional disposal methods and contributing to the sustainable production of valuable bioproducts such as biofuels, by releasing fermentable sugars [8,9,10], and animal feed, by improving protein availability [8,11]. Additionally, keratinases play a crucial role in the pharmaceutical industry by processing keratin-based biomaterials for the development of novel therapeutic products and drug delivery systems [12,13,14]. Recent advances in keratinase research have further highlighted their versatility and efficiency in transforming waste into useful resources, underscoring their importance in promoting sustainable practices [15,16,17,18,19,20,21].

Metagenomics has revolutionized microbiology and biotechnology by enabling the exploration of microbial diversity and the discovery of novel enzymes directly from environmental samples [22,23]. This approach circumvents the limitations of traditional culture-based methods, which can only cultivate a small fraction of microbial species [24,25]. By extracting and sequencing DNA from environmental samples, metagenomics provides insights into the genetic potential of entire microbial communities, including their enzymatic capabilities [26].

To enhance the efficiency and accuracy of keratinase discovery, we employed Hidden Markov Model (HMM)-based screening to detect potential keratinase genes. Recently, HMM has been widely used in functional macro genome screening and bioinformatics analysis [27,28,29,30], especially for protein secondary structure prediction studies [31,32]. Instead of relying on individual sequences to query a database, this approach uses a collection of previously validated sequences to construct a single probabilistic model for an entire gene family, resulting in accurate sequence matching and thus reducing the likelihood of false positives [33,34,35,36,37,38,39,40]. The HMM framework enables the construction of reliable probabilistic models for protein families, allowing for accurate modeling of active site-specific matches, insertions, and deletions [41,42,43,44]. Learning the secondary structures of proteins such as α-helices and β-sheets through HMMs and applying these HMMs to the large amount of unknown novel sequence data and structures of macrogenomes [45,46,47,48,49,50,51,52,53,54,55,56,57], and using the output probabilities of the HMMs for predicting the secondary structures of the sequences, has allowed for glimpses of unknown microbial biosynthetic potentials, leading to the discovery of more valuable proteins [32,58,59].

The increasing industrial and environmental demands for sustainable bioprocesses highlight the need for novel enzymes like keratinases, known for their versatility in waste degradation and bioactive compound synthesis [21,60,61]. However, their efficacy is often limited by substrate specificity, operational stability, and environmental adaptability. Our study leverages metagenomics to discover novel keratinases with improved and unique properties from the ESMatlas ESM Metagenomic Atlas database, bypassing traditional culturing limitations [55]. By focusing on candidates with structural similarity to known keratinases yet low DNA sequence similarity, and conducting motif alignment analysis, we aim to expand the keratinase repertoire and introduce enzymes with potentially unparalleled efficiency and specificity [62]. This endeavor could revolutionize various applications and advance our understanding of microbial biodiversity’s biotechnological potential.

Despite advancements in keratinase research, a significant gap remains in our understanding of the enzyme’s diversity and functionality in nature. Traditional discovery methods have focused on a limited range of cultivable microbial sources, neglecting the vast biodiversity in environmental samples [24,63]. This has left a large portion of the microbial and enzymatic landscape unexplored. Metagenomics has begun to reveal the genetic diversity in environmental niches, but its application for keratinase discovery is still emerging [26,64,65]. Our study aims to bridge this gap by employing metagenomic analysis to systematically explore environmental samples for novel keratinase genes, focusing on those with unique attributes. This approach seeks to expand the enzymatic toolkit for industrial and environmental applications and deepen our understanding of microbial biodiversity and its biotechnological implications.

In this study, two novel keratinases were discovered from the environmental database which harbors a rich, yet largely unexplored diversity of keratinase enzymes with unique properties, and can significantly enhance industrial and environmental processes. The aim is to uncover these hidden biocatalysts through metagenomic analysis of the ‘ESMatlas’ (ESM Metagenomic Atlas) database, focusing on enzymes with a high structural similarity to known keratinases, low DNA sequence similarity, and unique enzymatic properties suitable for industrial applications [34,35,40]. By expanding the understanding of keratinase diversity, this research seeks to contribute to the advancement of biotechnology and environmental sustainability [10].

## 2. Materials and Methods

### 2.1. Bioinformatics Analysis

To identify novel keratinase genes, a comprehensive bioinformatics approach was employed. The Esmatlas macrogenomic database, which compiles metagenomic data from environmental samples [66], served as the primary source for sequence retrieval. BLAST 2.13.0 (Basic Local Alignment Search Tool, Bethesda, MD USA) searches were conducted using keratinase sequences from the S8 family, including *Bacillus subtilis* and *Thermoactinomyces* keratinases, as query sequences [65,67,68]. Candidate genes were selected based on their sequence similarity to known keratinases, with a focus on those showing a low sequence identity, indicating potential novelty.

To identify conserved motifs and domains, HMMER was utilized for Hidden Markov Model-based searches, which revealed key regions, including active site residues and substrate-binding domains, characteristic of keratinases [31,32]. Multiple sequence alignments were then performed using Clustal Omega 1.2.4  [69], Muscle [70], and MAFFT [71], allowing for precise identification of conserved regions critical for enzyme function [72]. These alignments helped further refine the selection of candidate genes for downstream analysis.

Phylogenetic relationships of the keratinase genes were explored by constructing a tree with MEGA11, using neighbor-joining analysis, which confirmed that the identified genes, including *ker820* and *ker907*, belonged to distinct branches, suggesting their novelty [73]. In addition, sequences with low-quality regions were trimmed using TrimAL (version 1.5.0) to ensure high-quality alignment and modeling [74]. Structural predictions for the keratinases were generated using Modeller 10.5, based on homology with known keratinase structures [75]. The 3D models were visualized with PyMOL-3.1.5.1, ensuring structural integrity [76]. Structural alignment with known keratinases was performed using TM-align, and RMSD calculations confirmed the high similarity of the models [77]. The ESPript (version 3.0.10) tool was used to visualize conserved active site residues, such as Asp39, His72, Ser224, His397, and Glu315 in subtilisin BPN’ numbering., which were confirmed to be conserved [78].

To predict the functional potential of the novel keratinases, CLEAN (Contrastive Learning–Enabled Enzyme Annotation), a machine learning model based on contrastive learning, was used to assign EC numbers to the enzymes [79]. This method enhances the accuracy, reliability, and sensitivity of enzyme functional predictions, especially for previously uncharacterized enzymes, providing further insights into the enzymatic capabilities of ker820 and ker907.

To understand their properties, the ExPASy Compute pI/MW tool was used to calculate the isoelectric points (pI) and molecular weights (MW) of the keratinases [80,81,82,83].

### 2.2. Cloning and Heterologous Expression of Keratinase Genes

#### 2.2.1. Plasmid Construction and Cloning of Keratinase Genes

The *ker820* and *ker907* genes were synthesized by Novopro (Shanghai, China) based on the amino acid sequences predicted from computational analysis. The synthesized genes were cloned into the pGEX-4T-1 expression vector (Takara, Shiga, Japan) to facilitate the production of recombinant GST-tagged keratinases. The pET-28a(+) plasmids (Novopro, Shanghai, China), which contained the amino acid sequences of *ker820* and *ker907* predicted from the computational analysis, were used as templates for Gibson assembly based seamless cloning. Primers used for seamless cloning were designed to include overlapping sequences with the pGEX-4T-1 vector and are listed in Appendix A.2.

The ClonExpress II One Step Cloning Kit (Vazyme, Nanjing, China) was used to ligate the amplified DNA fragments into the linearized pGEX-4T-1 vector, followed by transformation into *E. coli* DH5α cells (Takara, Shiga, Japan) for cloning.

#### 2.2.2. Transformation and Co-Expression of Recombinant Keratinase Genes

Recombinant pGEX-4T-1-*ker820* and pGEX-4T-1-*ker907* plasmids were transformed into *E. coli* Rosetta (DE3) (Novagen, Madison, WI, USA) for heterologous expression. Transformed cells were cultured in LB medium supplemented with chloramphenicol (10 μg/mL) and ampicillin (100 μg/mL) (Sigma-Aldrich, St. Louis, MO, USA), together with L-arabinose (0.5 mg/mL) and tetracycline (5 ng/mL) for plasmid maintenance. When the culture reached an optical density at 600 nm (OD_600_) of 0.5, the expression of GST–keratinase fusion proteins was induced by adding isopropyl-β-D-1-thiogalactopyranoside (IPTG; Sigma-Aldrich, St. Louis, MO, USA) to a final concentration of 0.1 mM.

#### 2.2.3. Co-Expression with Molecular Chaperone

To enhance protein solubility, the pG-KJE8 molecular chaperone plasmid (Takara, Shiga, Japan) was co-transformed with pGEX-4T-1-*ker820* and pGEX-4T-1-*ker907* into *E. coli* Rosetta (DE3). The pG-KJE8 plasmid encodes a set of molecular chaperones that assist in the proper folding of recombinant proteins. The co-expression was performed under the same conditions as described above, with an additional induction of the chaperone expression using L-arabinose (0.5% final concentration; Sigma-Aldrich, St. Louis, MO, USA).

#### 2.2.4. Optimization of Expression Conditions

To optimize the expression of the recombinant keratinases, several factors were tested:

1. Induction Temperature:Protein expression was induced at temperatures of 16 °C, 20 °C, 28 °C, and 37 °C to determine the optimal conditions for soluble protein expression. Soluble expression was monitored using SDS-PAGE (Bio-Rad, Hercules, CA, USA).
2. IPTG Concentration: The effect of IPTG concentration on protein expression was evaluated by varying the IPTG concentration from 0.02 mM to 0.5 mM. Optimal conditions were selected based on the level of soluble protein expression.

#### 2.2.5. Protein Purification

After 8 h of incubation at 20 °C, the cells were harvested by centrifugation at 7000 rpm for 30 min at 4 °C (Beckman, Brea, CA, USA). The cell pellets were resuspended in equilibration buffer (50 mM NaH_2_PO_4_, 300 mM NaCl, pH 8.0) and subjected to sonication (Scientz, Ningbo, China) on ice to lyse the cells. The lysate was then clarified by centrifugation at 12,000 rpm for 10 min at 4 °C.

The recombinant GST-tagged keratinases were purified using GSH affinity chromatography with GST-tag Purification Resin (Beyotime, Shanghai, China), followed by elution with 10 mM GSH. The recombinant GST-tagged keratinases were purified using GSH affinity chromatography with GST-tag Purification Resin (Beyotime, Shanghai, China), followed by elution with 10 mM GSH. Protein purity was assessed by SDS-PAGE (Bio-Rad, Hercules, CA, USA), and the BCA protein assay kit (Thermo Fisher, Waltham, MA, USA) was used to determine the concentration of the purified enzyme.

#### 2.2.6. Evaluation of Expression and Purity

The soluble fractions of the induced cultures were analyzed by SDS-PAGE (Bio-Rad, Hercules, CA, USA), confirming the expression of GST-ker820 and GST-ker907 fusion proteins.

### 2.3. Enzymatic Characterization

#### 2.3.1. Enzymatic Activity Assays

The enzymatic activity of GST-ker820 and GST-ker907 was determined by measuring the hydrolysis of casein. The reaction was initiated by mixing 20 μL of recombinant enzyme solution with 40 μL of 2% casein solution, followed by incubation at 40  °C for 10 min.

The reaction was terminated with 40 μL of 0.4 M trichloroacetic acid (TCA), and the mixture was centrifuged. To the supernatant, 200 μL of 0.4 M ${\mathrm{Na}}_{2}$${\mathrm{CO}}_{3}$ and 40 μL of Folin–Phenol reagent were added. After incubation at 40  °C for 20 min, the absorbance of the resulting blue complex was measured at 680 nm. The amount of tyrosine released was quantified by comparing the absorbance values to a standard curve Figure A3 prepared from known tyrosine concentrations.

The enzyme activity was calculated using the following equation:

(1) $$\mathrm{Enzyme}\;\mathrm{Activity}\;\left(U/\mathrm{mg}\right)=\frac{M_{\mathrm{Tyr}}\times 0.1}{0.02\times 10\times C_{\ker}}$$

where:

$M_{\mathrm{Tyr}}$: Tyrosine equivalent produced (μmol)

0.1: Reaction volume (100 μL)

0.02: Enzyme volume (20 μL)

10: Reaction time (10 min)

$C_{\ker}$: Enzyme concentration (mg/mL)

This equation normalizes the enzymatic activity to units per milligram of enzyme, where one unit (U) is defined as the amount of enzyme that releases 1 μmol of tyrosine per minute under the assay conditions.

#### 2.3.2. Optimal Temperature and pH Determination

Optimal pH and temperature were determined by incubating 20 μL recombinant enzyme solution with 40 μL 2% casein solution in varying buffer conditions from pH 3.0 to 12.0 and temperatures from 30 to 80  °C.

#### 2.3.3. Thermal Stability Assay

Thermal stability was assessed by pre-incubating enzymes at 50  °C, 60  °C, and 70  °C for up to 120 min. Residual activity was measured at regular intervals by incubating the withdrawn aliquots with a casein solution, as described above.

#### 2.3.4. Effect of Metal Ions and Additives

Enzymes were pre-incubated with various metal ions (e.g., ${\mathrm{Mn}}^{2+}$, ${\mathrm{Ca}}^{2+}$, ${\mathrm{Fe}}^{3+}$), chelators (EDTA, EGTA), and surfactants (e.g., SDS) at concentrations of 1–10 mM. Relative activity was determined by incubating the withdrawn aliquots with a casein solution as described above and compared to a nonadditive control.

#### 2.3.5. Storage Stability Assay

Storage stability was evaluated by storing enzyme solutions at 4  °C and 25  °C, with activity measured at multiple time points over 10 days by incubating the withdrawn aliquots with the casein solution, as described above.

#### 2.3.6. Enzyme Kinetics and Lineweaver–Burk Plot

Kinetic parameters ($K_{m}$ and $V_{\max}$) were determined using casein and cat-hair powder as substrates across concentrations of 1–10 mg/mL. Initial velocities were calculated, and Lineweaver–Burk plots were used for linear regression analysis.

### 2.4. In Vitro Degradation of Keratin Substrates

To evaluate substrate specificity, keratin substrates including chicken feathers, cat hair, and human hair were used as substrates. Each substrate was washed thoroughly with warm water, rinsed with distilled water, and then autoclaved at 121  °C for 20 min to ensure sterilization. After autoclaving, the substrates were dried in an oven at 60  °C to a constant weight and cut into uniform 1 cm fragments prior to use.

Each 4 mL reaction mixture contained 1 mL of purified recombinant GST-keratinase (ker820 or ker907, 100 μg/mL) and 3 mL of Gly-NaOH buffer (pH 9.0). The substrates were incubated with the enzymes at their respective optimal temperatures under shaking conditions (200 rpm) for 3 days. The biodegradation rate of feathers was determined in terms of % weight loss after incubation with recombinant keratinases.

To quantify the degradation of keratin substrates, residual solid material was collected after incubation by filtration. The residues were washed thoroughly with distilled water to remove any loosely bound enzymes, peptides, or soluble degradation products. The washed residues were then dried in an oven at 60  °C until a constant weight was achieved. The dry mass was recorded using pre-weighed aluminum weighing boats. The degradation efficiency was calculated as the percentage of weight loss using the following equation:

(2) $$\mathrm{Substrate}\;\mathrm{Degradation}\;\left(\%\right)=\left(\frac{W_{\mathrm{initial}}-W_{\mathrm{residual}}}{W_{\mathrm{initial}}}\right)\times 100$$

where $W_{\mathrm{initial}}$ is the initial dry weight of the keratin substrate and $W_{\mathrm{residual}}$ is the dry weight of the substrate remaining after enzymatic treatment.

### 2.5. Amino Acid Analysis by Hplc

The degradation of feather powder by keratinases was evaluated by quantifying free amino acids released during hydrolysis. Reaction solutions collected before and after enzymatic degradation by ker820 and ker907 were first centrifuged at 13,000× *g* for 10 min at 4 °C, and the resulting supernatants were filtered through 0.22 µm syringe filters to remove insoluble residues. The filtrates were dried in a vacuum concentrator and derivatized with phenylisothiocyanate (PITC) to enhance detection sensitivity. After incubation with PITC at room temperature for 20 min, the samples were dried again and redissolved in 200 µL of solvent A (1.4 mM sodium acetate anhydrous, 0.1% triethylamine, and 6.0% acetonitrile, pH 6.1). The solutions were centrifuged at 13,000× *g* for 10 min at 4 °C, and the supernatants were analyzed using a reverse-phase HPLC system (LC20, Shimadzu, Kyoto, Japan) equipped with a C18 column (250 mm × 4.6 mm, 5 µm particle size).

### 2.6. Statistical Processing of Results

All quantitative experiments, such as enzymatic activity and stability assays, were performed in triplicate, and the results are presented as mean ± standard deviation (SD). Qualitative analyses, including structural modeling, were conducted once to obtain representative results. Data processing and plotting were carried out using GraphPad Prism 9 (GraphPad Software, San Diego, CA, USA). Linear regression analysis was applied and mean values were calculated for datasets with triplicate measurements.

## 3. Results

**Figure 1:**
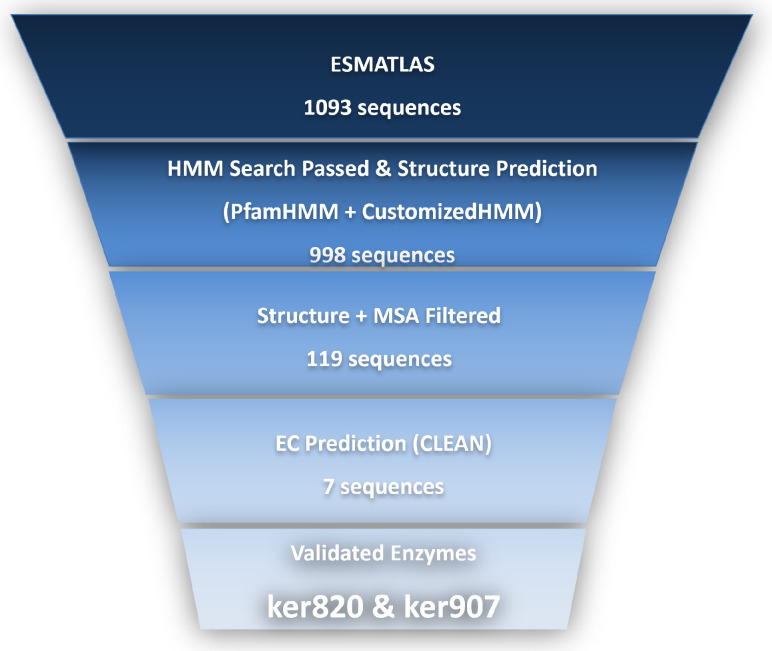
Workflow for the mining and characterization of novel keratinases from the Esmatlas macrogenomic database. A total of 1093 candidate sequences were initially retrieved and subjected to a multi-layered bioinformatics filtering strategy, including HMM profile searches, structural prediction, structural and sequence alignment, and EC number prediction using CLEAN. The sequences were narrowed to 7 high-confidence keratinase candidates. These genes were then cloned, heterologously expressed, and enzymatically tested, leading to the identification of two active keratinases, ker820 and ker907.

The overall experimental workflow for the identification and characterization of novel keratinases is summarized in Figure 1. The pipeline begins with metagenomic sequence mining and structural prediction, followed by cloning, heterologous expression, and purification of the target enzymes. Subsequent biochemical assays, including activity characterization and stability testing, were performed to evaluate the enzymatic properties of the candidates. This integrated strategy ensured that computational predictions were systematically validated through experimental approaches.

### 3.1. Identification of Novel Keratinase Genes

A total of 1093 candidate keratinase genes were initially retrieved from the Esmatlas metagenomic database using BLAST queries based on S8 family keratinases, such as *Bacillus subtilis* keratinase and *Thermoactinomyces* keratinase. These queries were selected based on their known keratinolytic activity, providing a strong basis for identifying homologous sequences in the Esmatlas database. To refine the selection, HMM-based screening was performed to identify conserved domains characteristic of keratinases. This analysis revealed the presence of key catalytic motifs typically associated with keratinase activity (e.g., Asp–His–Ser triad) in a subset of sequences.

The results of the HMM search and subsequent sequence alignment analysis are summarized in Table 1.

Among these, two genes (designated *ker820* and *ker907* Appendix A.1) were selected for further study. These sequences showed 45.5% and 43.2% identity, respectively, to known keratinases, indicating potential novelty. HMM analysis confirmed that both sequences matched the Peptidase S8 domain profile, including the catalytic triad residues (Asp, His, and Ser) essential for serine protease activity, indicating they belong to the serine protease family that includes keratinases. While these genes exhibited some sequence similarity to known keratinases, they also displayed significant divergence, suggesting that they may represent a previously uncharacterized family of keratinolytic enzymes with distinct properties.

**Figure A1:**
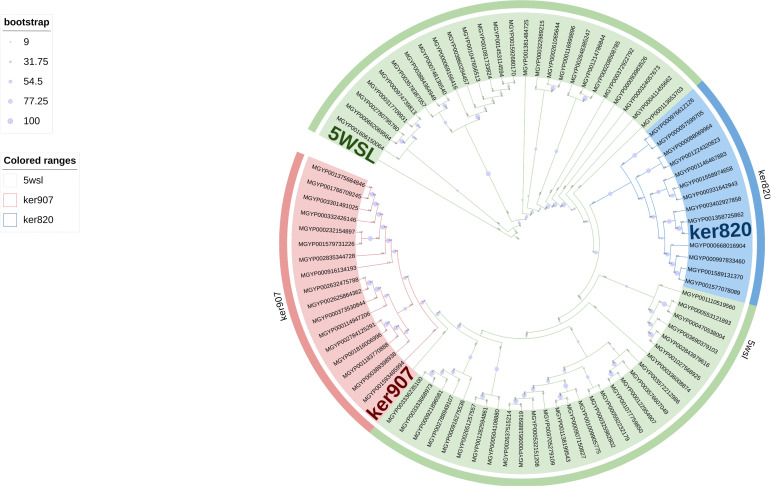
Phylogenetic analysis of keratinase candidate genes and reference sequences.The amino acid sequences were first aligned using MAFFT, and a maximum likelihood phylogenetic tree was generated using MEGA X with 1,000 bootstrap replicates. TM-align was employed to confirm structural similarity among selected sequences prior to tree construction. The tree was visualized in iTOL with color-coded clades to distinguish known keratinases genes and the novel candidates, including *ker820* and *ker907*, which are highlighted and occupy distinct branches, indicating their potential classification as new subgroups of keratinolytic enzymes.

Phylogenetic analysis using MEGA X showed that *ker820* and *ker907* clustered on distinct branches separate from previously characterized keratinases, indicating their evolutionary divergence (Figure A1). This branching pattern supports their classification as novel members of the keratinase family.

### 3.2. Sequence Analysis and Structural Prediction

Following the identification of candidate keratinase genes, *ker820* and *ker907* were examined for structural features relevant to keratinolytic function. Multiple sequence alignment revealed conserved catalytic motifs, including residues Asp32, His64, and Ser221 in subtilisin BPN’ numbering [84]; nucleophilic serine motifs [85]; and the oxyanion-hole [86,87,88], which are essential for substrate binding and hydrolysis, consistent with known serine proteases. Variations in non-catalytic regions indicated potential differences in substrate specificity and enzymatic performance.

**Figure 2:**
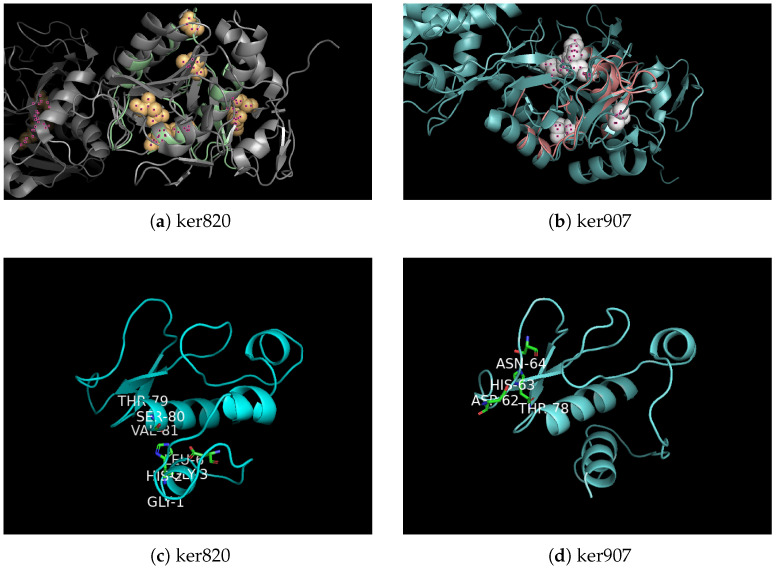
Structural modeling and comparison of novel keratinase candidates are shown in ball-and-stick representation. (**a**) Global structural alignment of ker820 with 5WSL. (**b**) Lobal structural alignment of ker907 with 5WSL. (**c**) Detailed comparison of predicted active sites of ker820 with conserved catalytic residues of 5WSL. (**d**) Detailed comparison of predicted active sites of ker907 with conserved catalytic residues of 5WSL.

Among several reference keratinases (5WSL, 7AJR, and 6FZX) used in the HMM search, the candidate sequences ker820 and ker907 were both identified within the 5WSL search results. Therefore, 5WSL, a well-characterized S8 family keratinase, was selected as the reference model for structural prediction and comparison. Homology modeling and structural comparison with the reference keratinase 5WSL (Figure 2) confirm that both enzymes possess the characteristic serine protease fold, supporting their classification as functional keratinases. In panel (a), the global structural alignment of ker820 with 5WSL shows an almost complete overlap of the core fold, corresponding to residues that are spatially superimposed between the two structures, indicating a high degree of structural conservation. The aligned active residues are highlighted in ball-and-stick representation, demonstrating precise correspondence within the catalytic site. Panel (b) displays a similar alignment for ker907, which again showing strong overlap of the conserved core fold. Panels (c) and (d) focus on the predicted active sites of ker820 and ker907, respectively, where the catalytic residues Asp–His–Ser are explicitly marked and closely coincide with those in 5WSL. In these figures, different colors are used solely to distinguish individual protein structures, while overlapping regions represent structurally conserved residues.

The structural alignment using TM-align revealed a high similarity between the predicted models and the reference keratinase structure (PDB ID: 5WSL), particularly around the catalytic triad residues (Asp–His–Ser), indicating that the overall fold and active site architecture were well conserved. For ker820, the RMSD values were 0.312 Å for the overall structure and 1.14 Å for the catalytic triad region, with an aligned length of 128.7 residues. Ker907 showed comparable results, with RMSD values of 0.315 Å for the overall structure and 0.285 Å for the catalytic triad region, and an aligned length of 132.8 residues. These low RMSD values, particularly within the catalytic regions, confirm the robustness of the homology models and support the classification of both enzymes as keratinases with conserved structural frameworks.

**Figure 3:**
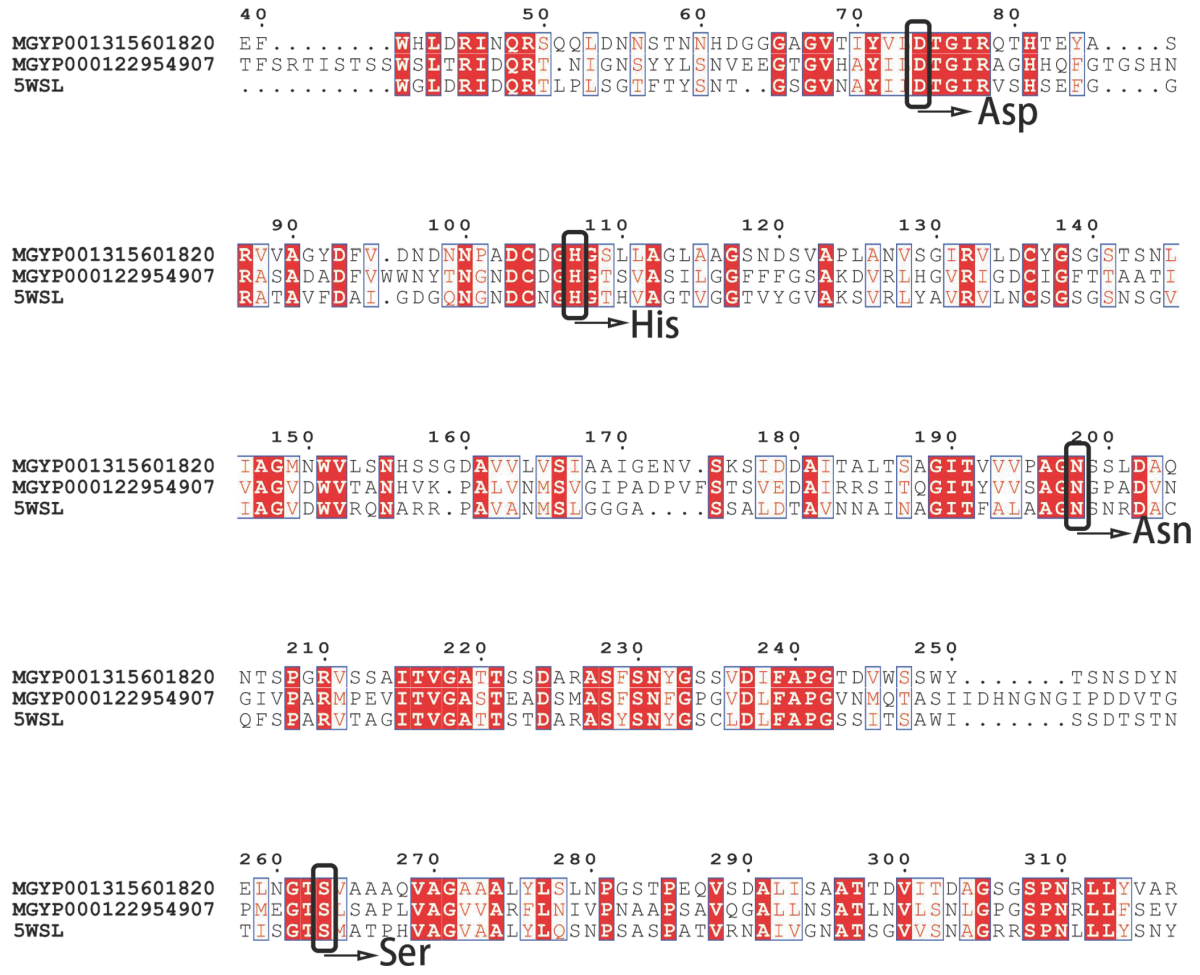
Multiple sequence alignment of keratinase candidates with reference keratinase 5WSL. Sequences were aligned using MAFFT and visualized with ESPript. Conserved catalytic residues (Asp, His, Ser, and Glu) and secondary structures are highlighted, supporting the functional prediction of ker820 and ker907.

Multiple sequence alignment (MAFFT, visualized with ESPript) in (Figure 3) with highlighted residues confirmed that *ker820* and *ker907* retain the canonical catalytic triad of subtilisin-like serine proteases. The catalytic residues are Asp74, His107, and Ser203 in *ker820*, and Asp163, His201, and Ser364 in *ker907*, corresponding to Asp32, His64, and Ser221 in subtilisin BPN’ numbering. These residues have been functionally validated in subtilisin-family proteases through site-directed mutagenesis, where the substitution of any one abolishes enzymatic activity  [84]. The nucleophilic serine motifs were identified as GTSGV (residues 200–204 in ker820) and GSGST (residues 361–365 in ker907), consistent with known subtilisin-like serine proteases [85]. In addition, both enzymes conserve the oxyanion-hole Asn198 (Asn155 in subtilisin numbering), which stabilizes the tetrahedral intermediate during catalysis and is likewise essential for activity  [86,87,88]. The alignment also revealed other conserved residues, such as His397 and Glu315, which are common in many keratinases but have not been directly confirmed as catalytically essential; these may contribute to structural stability or substrate positioning. Taken together, the conservation of experimentally validated catalytic residues strongly supports the classification of *ker820* and *ker907* as functional serine protease.

Additionally, physicochemical parameters (isoelectric points and molecular weights, using ExPASy Compute pI/MW) were predicted to provide a baseline characterization of ker820 and ker907 to confirm their consistency with known keratinases (Table 2). The predicted results showed ker820 (pI 8.92, 45.0 kDa) and ker907 (pI 7.86, 50.1 kDa) fall within the typical range for microbial keratinases [10]. Secondary structure predictions including proportions of α-helices, extended strands, β-turns, and random coils (Table 3) revealed that ker907 had a notably high proportion of random coils (46.30%), suggesting structural flexibility and potential broad substrate tolerance [89,90], while ker820 displayed a more balanced distribution of α-helices and extended strands, consistent with enhanced structural stability [91,92].

Functional annotation using the CLEAN tool assigned ker820 EC numbers 3.4.21.62 and 3.4.24.12, while ker907 was predicted as 3.4.21.62 (Table 2). Both enzymes were classified as serine endopeptidases, with ker820 also showing metalloproteases features, indicating their potential to hydrolyze peptide bonds in a broad range of substrates  [93]. These analyses served to verify that both ker820 and ker907 share the general physiclchemical properties of keratinases, rather than to suggest significant deviation from known examples.

Collectively, these findings confirm that both enzymes possess conserved active sites and structural frameworks typical of keratinases, supporting their potential for keratinolytic degradation.

### 3.3. Cloning and Expression of Keratinase Genes

**Figure 4:**
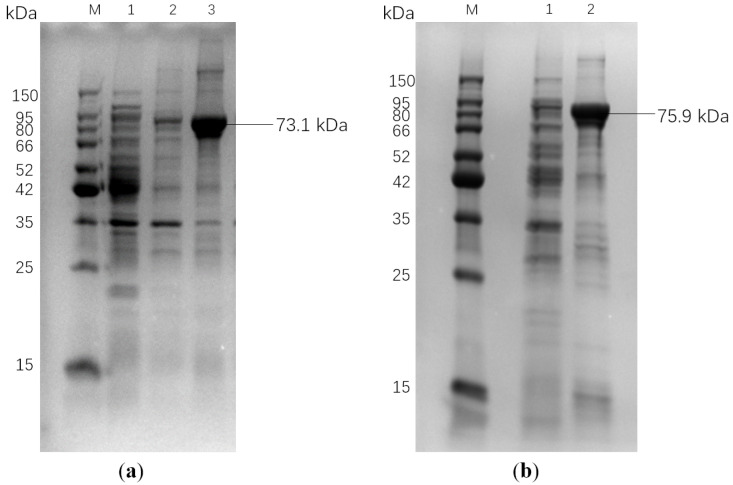
SDS-PAGE analysis of the recombinant keratinases GST-ker820 (**a**) and GST-ker907 (**b**) expressed in *E. coli* Rosetta (DE3). (**a**): M: protein marker; 1: background control; 2: chaperone control; 3: GST-ker820 (73.1 kDa); (**b**): M: protein marker; 1: background control; 2: GST-ker907 (75.9 kDa).

To further investigate the functional properties of the identified keratinase genes, *ker820* and *ker907* were cloned into the pGEX-4T-1 vector and heterologously expressed in *E. coli* Rosetta (DE3) cells with pG-KJE8 chaperone to improve solubility. SDS-PAGE analysis confirmed successful expression of both proteins, with clear bands at 73.1 kDa for GST-ker820 and 75.9 kDa for GST-ker907, corresponding to their expected molecular weights (Figure 4).

**Figure A2:**
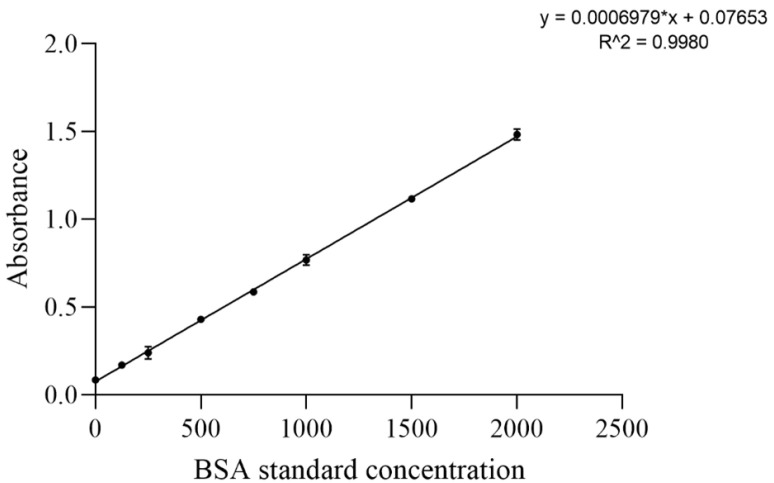
BCA standard curve (protein concentration determination).

**Figure A3:**
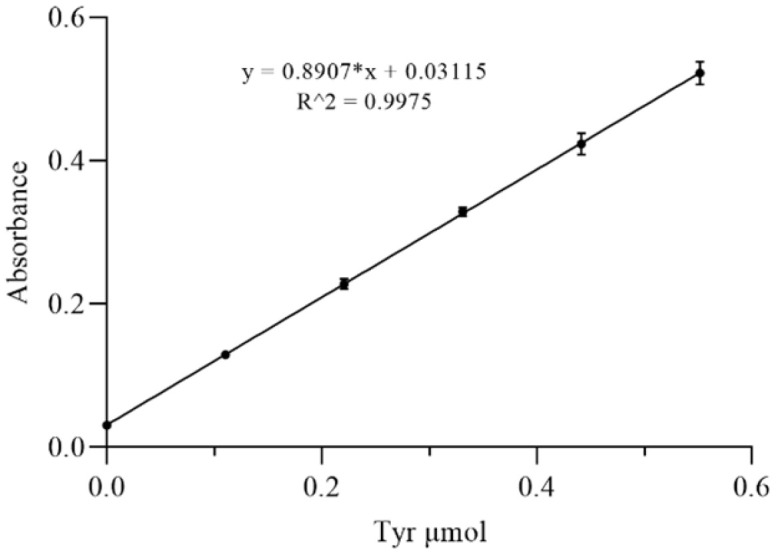
Tyrosine standard curve used for enzyme activity measurement.

Affinity purification yielded soluble and active enzyme preparations with approximately 10-fold purification and activity recovery exceeding 70% (Table 4). Protein concentrations were quantified using the BCA method (Figure A2). Importantly, comparison with pET-28a constructs demonstrated similar activity levels, indicating that the GST fusion tag did not affect catalytic function.

The purified enzymes were subsequently tested against keratinous substrates, including chicken feathers, cat hair, and human hair, confirming their ability to degrade insoluble keratin (details in the following section). These results demonstrate that both GST-ker820 and GST-ker907 were successfully expressed in soluble form and could be purified using GSH affinity chromatography and retain keratinolytic activity, thereby providing the basis for detailed biochemical characterization.

### 3.4. Biochemical and Kinetic Properties of New Keratinases

The enzymatic properties of recombinant ker820 and ker907 were characterized to determine their efficiency and suitability under different conditions. Key parameters included optimal temperature, pH, stability, substrate specificity, and kinetic constants.

#### 3.4.1. Optimal pH and Temperature

**Figure 5:**
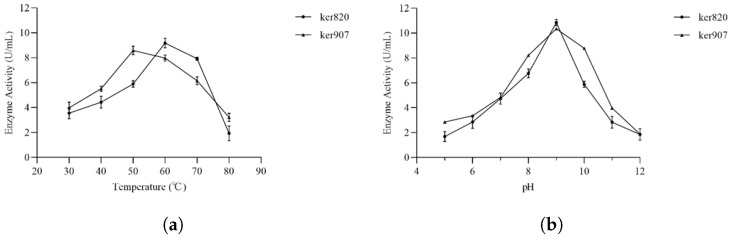
Temperature (**a**) and pH (**b**) dependence of enzymatic activity characterization for GST-ker820 and GST-ker907. The optimal pH was determined at pH ranging from 3.0 to 12.0. The optimal temperature was determined at temperatures ranging from 30 °C to 80 °C. Mean values are plotted with error bar representing the mean standard deviation of triplicates in each individual experiment. (**a**) Effect of temperature on the activity of GST-ker820 and GST-ker907. (**b**) Effect of pH on the activity of GST-ker820 and GST-ker907.

The effect of temperature on keratinase activity was examined at temperatures ranging from 20 to 80 °C (Figure 5). GST-ker820 displayed maximum activity at 60 °C, whereas GST-ker907 was most active at 50 °C. Activity declined sharply above these temperatures, indicating reduced activity at higher heat. Both enzymes therefore functioned optimally in the mesophilic to moderately thermophilic range, consistent with reported microbial keratinases [94].

The effect of pH was evaluated in the pH range of 4.0 to 10.0 (Figure 5). Both enzymes exhibited maximal activity at pH 9.0, with activity decreasing under more acidic or strongly alkaline conditions. This alkaline optimum is characteristic of keratinases and supports potential applications in keratin-rich industrial waste streams [94].

#### 3.4.2. Kinetic Parameters

To further characterize enzymatic activity, the kinetic parameters of GST-ker820 and GST-ker907 were determined using the Michaelis–Menten model. Substrate concentrations ranging from 0.1 to 10 mg/mL of casein and feathers were tested, and the resulting data were fitted to the Michaelis–Menten Table 5.

When using casein as the substrate, GST-ker820 exhibited a $K_{m}$ value of 9.81 mg/mL and a $V_{\max}$ of 120.99 U/mg, whereas GST-ker907 showed a $K_{m}$ of 5.25 mg/mL and a $V_{\max}$ of 89.52 U/mg. When using feathers as the substrate, the $K_{m}$ of GST-ker820 increased to 40.94 mg/mL with a corresponding $V_{\max}$ of 44.40 U/mg, while GST-ker907 demonstrated a $K_{m}$ of 21.04 mg/mL and a $V_{\max}$ of 19.89 U/mg. When compared with previously reported keratinases, ker820 and ker907 exhibited distinct kinetic profiles. IIP-K35 displays a similar $K_{m}$ for casein (9.8 mg/mL) to ker820 but a substantially higher $V_{max}$ (307.7 μmol/min), indicating a markedly greater catalytic turnover  [95]. RSA27 shows comparable substrate affinity ($K_{m}=5.6$ mg/mL) to ker907 but a higher catalytic rate ($V_{max}=142.4\;\mu$mol/min)  [96]. In contrast, US575 demonstrates a much lower $K_{m}$ (0.513 mg/mL) but also a lower $V_{max}$ (21.15 U/mg), suggesting strong substrate binding yet limited catalytic capacity  [97].

Overall, these comparisons reveal that ker820 and ker907 combine moderate substrate affinities with relatively high catalytic efficiencies. Specifically, ker820 is more effective for rapid substrate conversion, whereas ker907 exhibits stronger substrate binding, potentially favoring processes that require high specificity or sustained catalysis. These results suggest a stronger apparent affinity for soluble substrates such as casein compared to insoluble keratin. In addition, both keratinases possess distinct substrate specificities and a robust catalytic performance, making them promising candidates for industrial keratinolytic applications. In the subsequent stage of this study, ker820 and ker907 were further evaluated for keratinolytic activity under submerged fermentation using different keratin waste substrates.

### 3.5. Substrate Specificity

#### 3.5.1. Substrate Degradation

**Figure 6:**
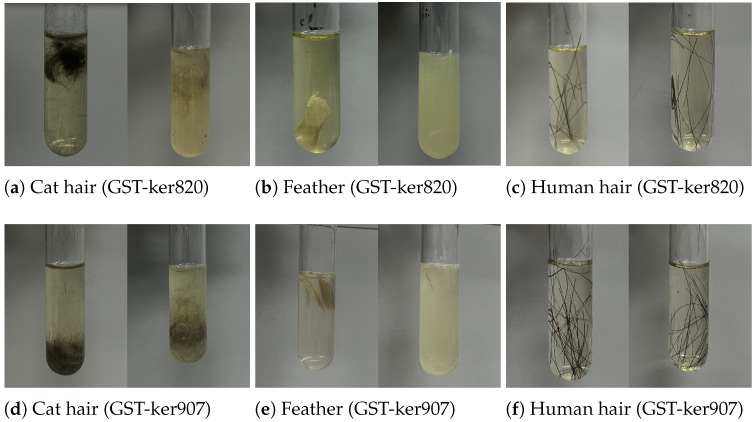
Visual assessment of keratin substrate degradation by recombinant keratinases. Cat-hair, chicken-feather, and human-hair samples were incubated with GST-ker820 (**a**–**c**) and GST-ker907 (**d**–**f**) for 3 days at their respective optimal temperatures. Significant degradation was observed for feather and cat hair, while human hair showed minimal changes.

The substrate specificity of recombinant keratinases was examined using chicken feathers, cat hair, and human hair as keratin-rich substrates. As shown in Figure 6, GST-ker820 and GST-ker907 efficiently degraded feather keratin within three days, leaving approximately 10% and 20% of solid feather mass, respectively. Cat-hair degradation was less pronounced, with  50% of the substrate remaining for GST-ker820 and  70% for GST-ker907 after the same period. Human hair proved highly resistant, with nearly 98% of the substrate persisting after enzymatic treatment. These results indicate that while both enzymes are highly active toward feather keratin, their efficiency diminishes against more compact substrates such as mammalian hair.

#### 3.5.2. Hplc Analysis of Hydrolysis Products

To further characterize the degradation process, the hydrolysates of feather powder were analyzed by high-performance liquid chromatography (HPLC) before and after enzymatic hydrolysis. As shown in Table 6 and Table 7, GST-ker820 released 20 amino acids after hydrolysis, whereas only 11 were detected before hydrolysis. Tyrosine, valine, arginine, isoleucine, proline, cystine, serine, and methionine were especially enriched, with valine, tyrosine, arginine, and isoleucine showing the most pronounced increases. Similarly, GST-ker907 generated 18 amino acids after hydrolysis, compared to 12 detected before hydrolysis, with isoleucine, tyrosine, arginine, leucine, alanine, proline, β-alanine, and glutamic acid notably elevated. Among these, isoleucine, tyrosine, arginine, and leucine were particularly abundant.

Together, these findings confirm that GST-ker820 and GST-ker907 effectively hydrolyze feather keratin and release a diverse profile of amino acids, validating their keratinolytic activity and highlighting their potential for valorizing keratin-rich waste.

### 3.6. Stability of Recombinant Keratinases

#### Thermal Stability

**Figure 7:**
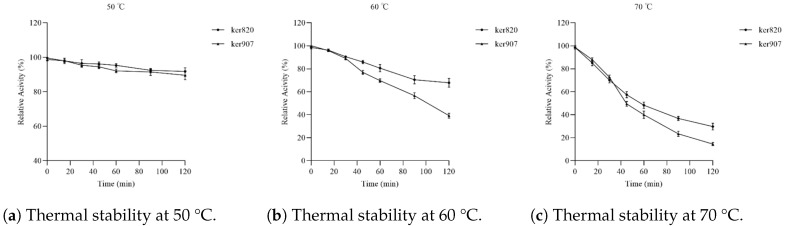
Thermal stability of recombinant keratinases GST-ker820 and GST-ker907. Enzymes were pre-incubated at 50 °C (**a**), 60 °C (**b**), and 70 °C (**c**) for 120 min. Residual activities were measured using casein as the substrate. Activity at 0 min was defined as 100%.

To evaluate the thermal stability of the keratinases, the enzymes were incubated at various temperatures (50 °C, 60 °C, and 70 °C) for extended periods (120 min). Enzyme activity was measured at each time point, and the results in Figure 7 revealed that the recombinant keratinases exhibited good thermal stability, with more than 90% of their activity retained after 120 min of incubation at 50 °C. GST-ker820 retained 70% of its activity after 120 min at 60 °C, exhibiting higher thermal stability compared to GST-ker907, which displayed less thermal stability of 50% activity retained under the same conditions. At higher temperatures, a gradual loss of activity was observed, with only around 40% of their original activity remaining at 70 °C. This indicates that the enzymes are moderately thermostable, which is advantageous for industrial applications requiring elevated temperatures. These results indicate the potential of GST-ker820 for high-temperature industrial applications.

### 3.7. Storage Stability

**Figure 8:**
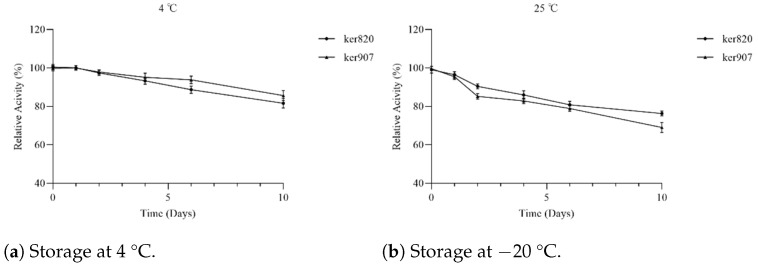
Storage stability of GST-ker820 and GST-ker907 over 10 days. Enzymes were stored at (**a**) 4 °C and (**b**) −20 °C. Residual activity was measured periodically using the casein–Folin assay, and activity at day 0 was defined as 100%.

The long-term stability of the keratinase enzymes was assessed by storing the purified proteins at 4 °C and −20 °C over a period of 10 days, as shown in Figure 8. Both enzymes showed good stability when stored at 4 °C. After 10 days of storage, GST-ker820 and GST-ker907 retained about 85% of their initial activity, suggesting that these enzymes could be stored at 4 °C for extended periods without significant loss of function. However, at −20 °C, the enzymes exhibited a slight decrease in activity after prolonged storage, where GST-ker820 kept about 75% of its initial activity, showing a relatively higher stability when stored in freezing conditions.

### 3.8. Effect of Metal Ions and Chemical Additives

The influence of various metal ions (Mn^2+^, Co^2+^, Ag^+^, Cu^2+^, Cr^2+^, K^+^, Ca^2+^, Mg^2+^, Li^+^, Zn^2+^, Ni^2+^, Fe^3+^) and chemical inhibitors (EDTA, EGTA) on the enzyme activity was also examined. The results in Table 8 revealed that GST-ker820 and GST-ker907 displayed enhanced or reduced activity in the presence of specific metal ions. For GST-ker820, Mn^2+^ and K^+^ significantly enhanced enzymatic activity, with Mn^2+^ leading to approximately a 60% increase and K^+^ resulting in a 40% increase relative to the control. Cu^2+^ and Fe^3+^ also showed a mild activating effect. In contrast, Co^2+^, Ag^+^, Cr^2+^, and Li^+^ strongly inhibited GST-ker820 activity. Notably, Cr^2+^ and Li^+^ almost completely abolished enzymatic activity, while Co^2+^ and Ag^+^ reduced the residual activity to 31% and 26%, respectively. Other ions, such as Ca^2+^, Mg^2+^, Zn^2+^, and Ni^2+^, exhibited negligible effects.

For GST-ker907, Ca^2+^ and Fe^3+^ were the most effective activators, enhancing activity by approximately 48% and 30%, respectively. Mn^2+^ and Zn^2+^ also slightly promoted activity. However, Co^2+^, Cr^2+^, and Li^+^ exerted inhibitory effects, with Cr^2+^ showing the strongest suppression, leaving only about 14% of residual activity. Other tested ions had minimal influence on GST-ker907.

Additionally, the presence of metal ion chelators EDTA and EGTA resulted in mild inhibition of both GST-ker820 and GST-ker907, although substantial enzymatic activity was retained, indicating that the enzymes are not strictly dependent on divalent metal cofactors for catalysis.

### 3.9. Inhibition Studies

To investigate potential enzyme inhibitors, the keratinases were incubated with various denaturants, including DTT, GuHCl, urea, and SDS. Results in Table 9 reveal that DTT and GuHCl slightly enhanced the enzyme activity, while urea caused moderate inhibition. SDS had minimal effect, indicating the enzymes’ tolerance to denaturing agents, which could be beneficial for industrial applications involving harsh conditions.

## 4. Discussion

The identification and characterization of the two novel keratinases, ker820 and ker907, from the Esmatlas macrogenomic database highlight the promise of bioinformatics-guided enzyme discovery for novel enzymes with broad implications in environmental sustainability and green chemistry. Our findings show that both ker820 and ker907 possess robust enzymatic properties, including high thermal stability and optimal activity under alkaline conditions, which are critical traits for industrial waste treatment applications. These enzymes’ ability to degrade substrates such as feathers and cat hair further shows their potential in sustainable recycling practices across industries like textiles, agriculture, and environmental management [8,9,10,98].

Compared with previously reported microbial keratinases from *Bacillus*, *Streptomyces*, *Aspergillus*, and *Corynebacterium* species, ker820 and ker907 demonstrated comparable or superior keratinolytic properties. Ker820 displayed optimal activity at 60 °C and pH 9.0, maintaining significant activity at elevated temperatures, surpassing the thermal stability of many *Bacillus* keratinases. Ker907 exhibited optimal activity at 50 °C and pH 9.0, slightly lower than ker820, but still within typical ranges for microbial keratinases [67,99,100,101,102,103,104,105]. Both enzymes effectively degraded both α-keratin (cat hair) and β-keratin (chicken feathers), indicating broader substrate specificity than some reported keratinases, such as those from *Aphanoascus keratinophilus*, which are more substrate-selective [106]. Favorable kinetic parameters ($K_{m}$ and $V_{\max}$) further emphasize their efficiency in keratin hydrolysis and their potential for industrial applications.

The integration of computational tools, particularly Modeller, played a vital role in accelerating the identification of these novel keratinases. By generating accurate three-dimensional models of the candidate enzymes, we were able to determine their catalytic sites and functional domains, providing a better understanding of their keratin-degrading potential. Modeller has proven invaluable in this context, as it offers a level of structural prediction accuracy that significantly outperforms traditional homology modeling [107]. Additionally, the use of CLEAN (Contrastive Learning–Enabled Enzyme Annotation) for functional annotation added a layer of confidence in the classification of these enzymes, allowing us to assign EC numbers with high reliability [79].

In parallel, traditional machine learning methods, such as Hidden Markov Models (HMMs), were employed to detect sequences with an underlying keratinolytic ability, further strengthening the functional classification of the enzymes [27,29,108]. The combination of these advanced computational tools not only accelerated the process of identifying and classifying the keratinases, but also provided valuable insights into their structural and functional properties.

Despite these promising results, there are several avenues for further investigation that could make this research more comprehensive. For example, experimental determination of the enzymes’ structures via X-ray crystallography or cryo-EM would confirm the active site conformation and substrate interactions. Although the substrate degradation assays were conducted under each enzyme’s determined optimal pH and temperature, further optimization on the physicochemical characteristics of individual keratin substrates may enhance degradation performance. Such substrate-specific optimization will be explored in future studies to better simulate industrial processing conditions. Future studies could also explore alternative heterologous expression hosts, co-expression strategies, or protein engineering approaches to enhance solubility, yield, and secretion efficiency. Expanding the range of tested keratin-rich substrates, such as wool, horns, or human hair, would provide a more complete profile of substrate versatility. In addition, assessing long-term operational stability under repeated cycles, high substrate loads, or in the presence of industrial additives would provide practical insights into enzyme durability.

Looking forward, future research can enhance this approach by incorporating more advanced AI-driven methods to optimize the expression and stability of recombinant keratinases. Tools like ProteinMPNN, which use graph neural networks to optimize protein sequences for improved solubility and folding [109], offer an exciting avenue for improving the practical applicability of these enzymes. Moreover, the integration of solubility prediction models such as SoluProt, DeepSol, and ProSol could provide a more systematic way to select or engineer keratinase variants with a higher expression efficiency and stability in heterologous hosts [110,111,112].

Ultimately, these advancements in AI-driven protein design and solubility prediction will enable the rapid development of more novel efficient and robust biocatalysts, such as keratin waste valorization, and provide potential solutions for broader environmental challenges.

## 5. Conclusions

This study introduces a promising strategy for the identification and characterization of novel keratinases using bioinformatics and AI-driven tools. The integration of computational approaches, including Modeller, CLEAN, and HMMs, significantly accelerated the discovery of keratinases with desirable enzymatic properties, such as high thermal stability, alkaline activity, and substrate specificity for keratin degradation. These findings demonstrate the efficiency of combining metagenomic screening with AI-driven protein design in enzyme discovery.

In future research, the continued integration of advanced AI technologies, such as ProteinMPNN and solubility prediction tools, can further enhance the soluble expression and stability of recombinant keratinases, ultimately accelerating the development of robust biocatalysts for industrial and environmental applications. The methodology developed in this study not only contributes to the valorization of keratin waste, but also has broader implications for enzyme discovery in other environmental remediation efforts, providing a scalable, eco-friendly solution to a growing global challenge.

## Figures and Tables

**Table 1:** HMM search and sequence alignment results of candidate keratinase genes.

Gene	Gene Length (bp)	UniProt ID	Identity (%)	Similarity (%)	E-Value
*ker820*	445	A0A2H4A2Y5	62	45.5	$2.1\times 10^{-6}$
*ker907*	473	A0A2H4A2Y5	62	43.2	$3.4\times 10^{-5}$

**Table 2:** Predicted physicochemical and functional properties of potential keratinases.

Keratinase	Isoelectric Point (pI)	Molecular Weight (kDa)	Predicted E.C. Number (s)	Confidence Level
ker820	8.92	45.0	3.4.21.62	High
			3.4.24.12	
ker907	7.86	50.1	3.4.21.62	Medium

**Table 3:** Secondary structure analysis of amino acid sequences of potential keratinases.

Keratinase	α-Helix	Extended Strand	β-Turn	Random Coil
ker820	26.01%	24.94%	5.62%	43.43%
ker907	24.95%	22.20%	6.55%	46.30%

**Table 4:** Enzyme activity before and after purification (showing enzyme activity recovery for pET-28a(+)-ker820, ker907, and pGEX-4T-1-ker820, ker907).

Enzyme	Unpurified	Purified	Recovery
pET-28a(+)-ker820	1.384 U/mL	15.868 U/mL	91.64%
pET-28a(+)-ker907	1.365 U/mL	12.737 U/mL	81.32%
pGEX-4T-1-ker820	1.772 U/mL	15.213 U/mL	73.91%
pGEX-4T-1-ker907	1.068 U/mL	11.684 U/mL	89.31%

**Table 5:** Kinetic parameters of recombinant keratinases with casein and feather substrates.

Enzyme	Substrate	$K_{m}$ (mg/mL)	$V_{\max}$ (U/mg)
GST-ker820	Casein	9.81	120.99
	Feather	40.94	44.40
GST-ker907	Casein	5.25	89.52
	Feather	21.04	19.89

**Table 6:** Amino acid composition of feather degradation products by GSTker820.

Amino Acid	After Hydrolysis (mg/mL)	Before Hydrolysis (mg/mL)
Serine	0.0251	0.0011
α-Aminoadipic acid	0.0024	-
Glycine	0.0232	-
L-Alanine	0.0464	0.0031
Cystine	0.0374	-
Methionine	0.0087	-
Valine	0.4027	0.0012
Isoleucine	0.0913	0.0060
Leucine	0.0618	0.0009
Tyrosine	0.3940	0.0023
Phenylalanine	0.0362	0.0017
β-Alanine	0.0567	0.0013
γ-Aminobutyric acid	0.0045	-
Histidine	0.0319	-
Proline	0.0529	-
Tryptophan	0.0032	-
3-Methyl-L-histidine	0.0082	0.0006
Ornithine	0.0033	-
Lysine	0.0212	0.0003
Arginine	0.1825	0.0030

**Table 7:** Amino acid composition of feather degradation products by GSTker907.

Amino Acid	After Hydrolysis (mg/mL)	Before Hydrolysis (mg/mL)
Aspartic acid	0.0013	0.0008
Threonine	0.0482	-
Glutamic acid	0.0465	-
Serine	0.0059	-
Glycine	0.0201	0.0012
L-Alanine	0.0629	0.0027
Cystine	0.0224	-
Isoleucine	0.8450	0.0018
Leucine	0.0723	0.0006
Tyrosine	0.3218	0.0012
Phenylalanine	0.0295	0.0013
Lysine	0.0110	0.0003
β-Alanine	0.0473	0.0023
3-Methyl-L-histidine	0.0017	0.0006
Ornithine	0.0021	-
Proline	0.0451	-
Arginine	0.1063	0.0022
Proline	0.0536	0.0028

**Table 8:** Effect of metal ions and inhibitors on enzyme activity.

Ions	Relative Activity of GST-ker820 (%)	Relative Activity of GST-ker907 (%)
None	100.00	100.00
Mn^2+^	161.25 ± 1.57	123.89 ± 1.25
Co^2+^	31.45 ± 3.14	49.24 ± 2.05
Ag^+^	26.17 ± 1.52	63.52 ± 2.86
Cu^2+^	126.87 ± 2.31	101.45 ± 2.76
Cr^2+^	7.58 ± 2.25	14.12 ± 1.34
K^+^	141.26 ± 2.06	98.17 ± 2.38
Ca^2+^	87.72 ± 2.71	148.70 ± 2.56
Mg^2+^	71.34 ± 1.54	64.27 ± 2.01
Li^+^	7.12 ± 3.02	45.13 ± 1.34
Zn^2+^	63.17 ± 0.91	121.81 ± 0.95
Ni^2+^	97.24 ± 1.75	104.12 ± 1.57
Fe^3+^	117.45 ± 1.92	130.68 ± 3.21
EDTA	71.49 ± 2.13	87.01 ± 1.38
EGTA	75.81 ± 1.92	79.52 ± 1.41

**Table 9:** Effect of denaturants on enzyme activity.

Denaturants	Relative Activity of GST-ker820 (%)	Relative Activity of GST-ker907 (%)
DTT	107.26 ± 1.94	102.87 ± 0.76
GuHCl	103.89 ± 2.48	110.75 ± 1.84
Urea	86.25 ± 2.05	77.87 ± 0.97
SDS	104.75 ± 2.16	102.28 ± 2.06

**Figure A4:**
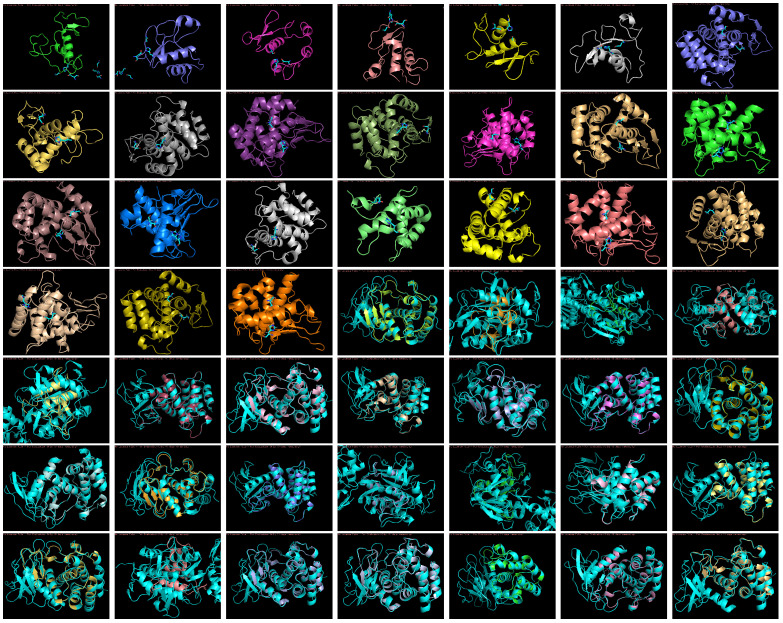
Structural alignments of keratinases with the reference structure using TM-align.

## Data Availability

The original contributions presented in the study are included in the article; further inquiries can be directed to the corresponding authors.

## Abbreviations

    The following abbreviations are used in this manuscript:

BLAST	Basic Local Alignment Search Tool
HMM	Hidden Markov Model
EC	Enzyme Commission (number)
RMSD	Root Mean Square Deviation
MAFFT	Multiple Alignment using Fast Fourier Transform
MEGA X	Molecular Evolutionary Genetics Analysis X
CLEAN	Contrastive Learning–Enabled Enzyme Annotation

## Appendix A

### Appendix A.1. Seuqneces of Novel Keratinases

>MGYP001315601820

SGQNGGSTGVWSLEVLDPGGAIPAPQGYWAPVPSSNNGEFWHLDRINQRSQQLDNNSTNNHDGGGAGVTIYVLDTGIRQTHTEYASRVVAGYDFVDNDNNPADCDGHGSLLAGLAAGSNDSVAPLANVSGIRVLDCYGSGSTSNLIAGMNWVLSNHSSGDAVVLVSIAAIGENVSKSIDDAITALTSAGITVVVPAGNSSLDAQNTSPGRVSSAITVGATTSSDARASFSNYGSSVDIFAPGTDVWSSWYTSNSDYNELNGTSVAAAQVAGAAALYLSLNPGSTPEQVSDALISAATTDVITDAGSGSPNRLLYVARPDIAITSDVSTLSAGETATLTFTLSDPSTDFIESDVSVSGGFLSEWNPVSSTSYAATFTPAVNSTNDGLISVATGGFSNSWGNTNADGSDSNNSITLTIDTSAPSAPSSLTTSSTTSDKTPTITGTAE

>MGYP000122954907

MKTHSRLLIAILITLAVVPSLAWHTGSAQNNQQEARGKDQEKFRKSKKPVREQYIVVLNSETSADEVEPLANEFLGKHGGTARHIYKHAIKGFSIQLPEPAAMALSRDPRVAYVEEDTEATFSRTISTSSWSLTRIDQRTNIGNSYYLSNVEEGTGVHAYILDTGIRAGHHQFGTGSHNRASADADFVWWNYTNGNDCDGHGTSVASILGGFFFGSAKDVRLHGVRIGDCIGFTTAATIVAGVDWVTANHVKPALVNMSVGIPADPVFSTSVEDAIRRSITQGITYVVSAGNGPADVNGIVPARMPEVITVGASTEADSMASFSNFGPGVDLFAPGVNMQTASIIDHNGNGIPDDVTGPMEGTSLSAPLVAGVVARFLNIVPNAAPSAVQGALLNSATLNVLSNLGPGSPNRLLFSEVRHGFLGQQIRSIGEGSTNVDSGVFLNPGQWLAMSGIGEIWAGVPFTSNNGPQDRK

### Appendix A.2. Primers Designed for Seamless Cloning

ker820-F: 5’-CGCGTGGATCCCCGGAATTCATGAACCACAAAGTACATCATCACCAT-3’
ker820-R: 5’-TCACGATGCGGCCGCTCGAGCTCGAGTTATTCAGCGGT-3’
ker907-F: 5’-CGCGTGGATCCCCGGAATTCATGAACCACAAA-3’
ker907-R: 5’-TCACGATGCGGCCGCTCGAGCTCGAGTTATTT-3’
